# The influence of addition of cranberrybush juice to pear juice on chemical composition and antioxidant properties

**DOI:** 10.1007/s13197-018-3233-8

**Published:** 2018-07-16

**Authors:** Sabina Lachowicz, Jan Oszmiański

**Affiliations:** Department of Fruit, Vegetable and Plant Nutraceutical Technology, Wrocław University of Environmental and Life Science, 37, Chełmońskiego Street, 51-630 Wrocław, Poland

**Keywords:** *Viburnum opulus*, *Pyrus communis* L., Antioxidant properties, Polyphenolic compounds, Functional fruit juice

## Abstract

The aim of this study was to verify whether interactions between bioactive compounds play an important role in the creation of functional fruit juices. Assessment of organoleptic parameters, colour, total soluble solids (TSS), total titratable acidity (TTA), antioxidant activity, polyphenols and vitamin C content and the interaction factor was performed. The juices were analysed before and after 5 months of storage at 25 °C. The effect of different dose mixtures on the quality of pear and cranberrybush juice was observed. The degree of consumer acceptance of the mixture of juices and the ratio TSS/TTA, which influence the preferences of consumers, were higher in the case of products PC1 and PC2. The analysed products differed significantly in the content of bioactive compounds, the highest content of phenolic compounds being observed for PC5. Taking into account the analysis of the interaction between juices, the most active was the PC2 juice.

## Introduction

The current trend of a healthy lifestyle has had an impact on the change in nutritional needs and growth of interest in functional foods. A varied and properly balanced diet and increasing physical activity are among the most important factors that affect the development of health and mental and physical well-being of consumers (Jiménez-Colmenero et al. 2001; Charalampopoulos et al. 2002; Granato et al. 2010). More and more often consumers choose those natural products with sensory advantages and heightened pro-health value (Layman 2014; Falguera et al. 2012). In order to meet customer demand, the food industry has begun to produce functional foods, which play a special role (Menrad 2003; Andrés et al. 2015). Functional food refers to food products enriched with raw materials exhibiting high value for health prevention, with characteristics intermediate between medicine and food. They are similar to traditional products, which are part of a normal diet (Hardy 2000; Gupta et al. 2015). A vital role is fulfilled by products rich in biologically active compounds, such as functional drinks. Functional beverages and juices are commonly consumed around the world and are located in the dynamically growing segment of functional foods. Consumers mainly in developed countries such as the United States, Canada and Europe have increased interest in natural and minimally processed vegetable drinks. A good example of such drinks is mixing different fruits without the addition of synthetic substances, which allows for attractive products with the content of bioactive substances. This may be important in processing fruits that are low in health-promoting compounds (Mena et al. 2011; Yong et al. 2009; Keenan et al. 2012; Teleszko and Wojdyło 2014).

Fruit and vegetables are characterized by high amounts of bioactive compounds. Among them, phenolic compounds display a wide range of properties that bring health benefits. There is considerable evidence supporting intake of vegetables and fruits rich in antioxidants which protect against diseases and oxidative damage. Therefore, increasing attention in the scientific community is devoted to the antioxidant activity in beverages and foods (Durak et al. 2015; Pérez-Jiménez and Saura-Calixto 2015; Zhang and Tsao 2016; Catel-Ferreira et al. 2015; Lachowicz et al. 2017a). The beverage compositions of different fruits can have different antioxidant capacities and abilities to scavenge oxygen free radicals. Beverages with tart and sweet fruit may have greater biological activity than one with single composition (Seymour et al. 2008; Will et al. 2008). The difference in the antioxidant activity may be due to the different content and diversity of polyphenol compounds. This phenomenon can be explained by the several kinds of interactions, either synergistic (at target sites of action) or antagonistic (inhibitory of action) (Kirakosyan et al. 2010). Gawlik-Dziki (2012) proposed a simple and effective method for determination of interactions between components in a mixture [as the interaction factor (IF)]. The IF value shows the strength of the interaction between compounds in a mixture.

Thus, the aim of this study was to verify whether interactions between bioactive compounds play an important role in the creation of functional fruit juices, and also to determine sensory attributes of pear and cranberrybush juice and the physicochemical properties such as colour, content of sugar, vitamin C, total soluble solids, antioxidant activity, and polyphenol content after processing and after a 5-month storage time at 20 °C in appropriate proportions (97.5/2.5, 95/5, 92.5/7.5, 90/10, and 75/25).

## Materials and methods

### Reagents and standards

ABTS (2,2′-azinobis-(3-ethylbenzothiazoline-6-sulphonic acid), Trolox (6-hydroxy-2,5,7,8-tetramethylchroman-2-carboxylic acid), TPTZ (2,4,6-tri(2-pyridyl)-s-triazine), acetic acid and, Folin-Ciocalteu reagent and methanol were purchased from Sigma-Aldrich (Steinheim, Germany). Sodium carbonate was purchased from Archem (Kamieniec Wroclawski, Poland).

### Plant materials

Fruit of cranberrybush (*Viburnum opulus)* cv. ‘Compactum’ (~ 3 kg) was obtained from a horticultural farm in Tymbark, Poland (49°43′45″N 20°19′27″E). Fruit of pears (*Pyrus communis* L.) cv ‘Konferencja’ (~ 3 kg) was obtained from horticultural from the Research Institute of Horticulture in Skierniewice, Poland (51°55′24″N, 020°5′58″E).

### Juice production

The process of production of juice from pears and cranberrybush involved three main technological steps:

1. Processing of pear juice. The pears were ground in a Thermomix appliance (Vorwerk, Wuppertal, Germany) (appliance with 1% solution of ascorbic acid—1 kg of fruit), then the pulps was pressed on a hydraulic press (SSRE, Warsaw, Poland) (at a piston thrust of 15 tons of pressure for 2 min) to obtain juice.
2. Processing of cranberrybush juice. The same procedure was repeated during the production of *Viburnum* juice (without the addition of ascorbic acid—to prevent enzymatic browning).
3. Juices from pears and cranberrybush, immediately after being obtained, were mixed in the proportions 97.5/2.5, 95/5, 92.5/7.5, 90/10, and 75/25, respectively. The smaller addition of cranberrybush to pear juices results from its characteristic strong taste and smell, which described by Sedat Velioglu et al. (2006).

Then, the juice products were pasteurized by heating to 100 °C for 5 min and put into glass jars, pasteurised (10 min), and cooled to 20 °C. Finally, seven different juices were obtained (Table 1). Each sample was prepared in two replicates. The products were analysed immediately after processing and after 5 months of storage at 25 °C (Wojdyło et al. 2014).

**Table 1:** The resulting products

No	Symbols	Product^a^
1	P1	100% PJ
2	PC1	97.5% PJ ÷ 2.5% CSJ
3	PC2	95% PJ ÷ 5% CSJ
4	PC3	92.5% PJ ÷ 7.5% CSJ
5	PC4	90% PJ ÷ 10% CSJ
6	PC5	75% PJ ÷ 25% CSJ
7	C1	100% CSJ

P1, juice from pear; C1, juice from cranberrybush

^a^The percentage share of the individual components was expressed in %

### Consumer evaluation

The sensory properties of juices obtained from pears and cranberrybush were evaluated using a 5-degree hedonic scale with boundary indications: ‘I do not like it very much’—‘I like it very much’. The assessment included the following quality attributes: taste, aroma, colour, consistency, and general assessment. It was conducted by a group of 15 consumers panellists. Coded samples were provided to the panellists for the evaluation at 20 °C in uniform 50-ml plastic containers (Lachowicz et al. 2017a).

### Chemical analyses

The basic parameters of the chemical composition—total soluble solids and vitamin C—were determined in juices according to Polish standards (PN-A-04019:1998; PN-90/A-75101/04). Results are reported as the arithmetic mean of three independent repetitions, taking into account the standard deviation (SD).

### Sugar analysis by the HPLC-ELSD

An analysis of sugar by the HPLC-ELSD method was performed according to the protocol described by Oszmianski and Lachowicz (2016) and Lachowicz et al. (2017b). All measurements were repeated three times. The results were expressed as mg per 100 ml FM.

### Colour parameters

Colour parameter (L*, a*, b*) of juices from pears and cranberrybush were determined by reflectance measurement with a Colour Quest XE Hunter Lab colorimeter. The samples were determined according to the method described by Wojdyło et al. (2014) and Šumić et al. (2013). Samples were measured against a white ceramic reference plate (L* = 93.92; a* = 1.03; b* = 0.52). The data were the mean of three measurements.

### Total polyphenol

The solvent for analysis was prepared and described previously by Lachowicz et al. (2017c). Total polyphenols were determined by the Folin-Ciocalteu method (Xiangqun et al. 2000). An aliquot (100 μl) of juices was mixed with 2000 μl of distilled water and 200 μl of Folin-Ciocalteu phenol reagent. Two hundred microlitres of sodium carbonate solution (200 g/L) was added to the mixture. The mixture was incubated at 20 °C for 1 h in darkness. Solutions of gallic acid from 0 to 500 mg/L were measured with the same procedure, for the creation of the calibration curve. Total polyphenolics were expressed as milligrams of gallic acid equivalents (GAE) per 100 ml.

### Antioxidant activity

The solvent for analysis was prepared and described previously by Lachowicz et al. (2017d). The ABTS°^+^ and FRAP assay were determined as previously described by Re et al. (1999) and Benzie and Strain (1996), respectively. Determinations by ABTS and FRAP methods were performed using a UV-2401 PC spectrophotometer (Shimadzu, Kyoto, Japan). The antioxidant activity was expressed as millimoles of Trolox per 100 ml. Percentage inhibition of the ABTS + radical was then calculated according to the method described by Durak et al. (2015). All assays were performed in triplicate.

### Theoretical approach

In accordance with the definition, the half-maximal inhibitory concentration (IC_50_) is a measure of the effectiveness of inhibitors. It is commonly used as a measure of antagonist drug potency in pharmacological research. The IC_50_ value is reliable for determining the activity of a single or two-compound mixture (e.g. isobolographic analysis) (Williamson, 2001). Further, the EC_50_ index quantitatively measures the amount of extractor extracts mixture that is required to exhibit half of the measured activity.

The following factor was also determined according to the method described by Gawlik-Dziki (2012) and Durak et al. (2015): the interaction factor (IF), which provides an explanation for the mode of interaction:

$$ {\text{IF}} = {\text{A}}_{\text{M}} / {\text{A}}_{\text{T}} $$

where, *A*_*M*_ = measured activity of a mixture of samples, and *A*_*T*_ = theoretically calculated mixture activity (based on the dose response of single components at various concentrations).

### Statistical analysis

Results were presented as mean ± standard deviation of three independent determinations. All statistical analyses were performed with Statistica version 12.5 (StatSoft, Krakow, Poland). Significant differences (*p *≤0.05) between means were evaluated by one-way analysis of variance (ANOVA) by Duncan’s multiple range test.

## Results and discussion

### Consumer evaluation of the tested products

**Fig. 1:**
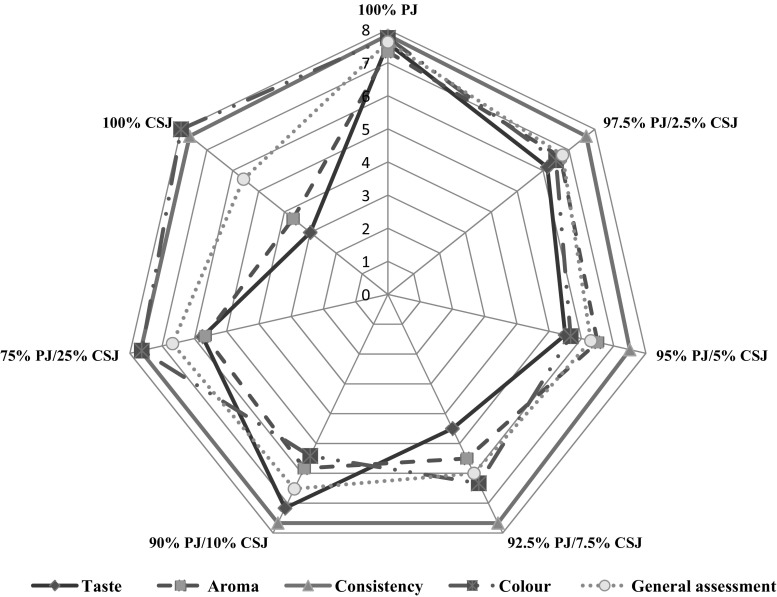
Consumer evaluation of analysed juices

Results of sensory evaluation of juices with pear and cranberrybush according to complex properties—taste, aroma, colour, consistency, and general assessment—are presented in Fig. 1.

Generally, the results showed that all juices were attractive in terms of consistency (≥ 7.5). Similar scores for colour were obtained for the cranberry juice, at around 8.00 (C1), pear juice, around 7.70 (P1), and with 50% addition of cranberrybush, around 7.63 (CP5). In turn, according to consumers, juices with 2.5 and 10% addition of cranberrybush (CP1 and CP3) had the lowest evaluation of colour (respectively, 5.67 and 5.42). The highest evaluation of taste of the products was obtained by P1 (around 7.58), CP1 (7.17) and CP2 (6.5). The lowest evaluation of taste, according to consumers, was obtained by C1 (3.00) and CP% (4.50). The highest scores of aroma were obtained for P1 (7.33), CP1 (6.67) and CP2 (6.50), while the lowest score was obtained for C1 (3.67). Only in three types of juices was aroma evaluation < 6: with 7.5% addition of cranberrybush (CP3) (5.50), 10% cranberrybush (CP4) (5.83) and 25% cranberrybush (CP5) (5.67) (Fig. 1).

Higher evaluation of products was obtained for pear juice (P1) and juices enriched with 2.5, 5, 7% addition of cranberrybush (respectively, CP1, CP2, CP3). These products were more attractive and accepted by the consumer. In turn, definitely unacceptable were cranberrybush juices (C1) and pear juice with 50% addition of cranberrybush (CP5). A larger proportion of cranberrybush worsens the taste of the juice. It is common knowledge that cranberrybush is characterized by specific bouquet of flavour. The acrid, bitter taste and specific and unpleasant smell of cranberrybush fruit are not acceptable for the consumers. Česonienė et al. (2012) confirmed that cranberrybush juice is not suitable for consumption raw. On the other hand, *Viburnum opulus* fruit is very good for processing, with a high content of bioactive compounds, which may be added in small amounts, enriching the quality of the final products (Sedat Velioglu et al. 2006). Sweet products with addition of a small amount of cranberrybush juice were characterized by the highest marks among the consumers, whereas the acidic and bitter ones were less accepted. Such relation has been confirmed by Teleszko and Wojdyło (2014), who reported that the taste plays an important role in the sensory evaluation of foods.

### Chemical composition

The highest content of vitamin C before storage was determined in C1 (36.78 g/100 ml) and was 16 times higher than P1 (Tables 2, 3). Generally, juices with the highest addition of cranberrybush juice showed higher concentration of vitamin C. After storage the content of vitamin C was 1.8 times lower than juices before storage.

**Table 2:** Effect of different dose of pear and cranberrybush juice, total soluble solids (TSS), vitamin C and total titratable acidity (TTA)

Samples	Immediately after processing				After storage 5 m at 25 °C			
	VIT C^a^	TTA^b^	TSS (°Brix)^c^	Ratio (TSS/TTA)	VIT C^a^	TTA^b^	TSS (°Brix)^c^	Ratio (TSS/TTA)
P1	2.41 ± 0.02d^‡^	0.66 ± 0.00c	14.00 ± 0.40	21.21	1.31 ± 0.01de	0.60 ± 0.00d	13.40 ± 0.21	22.33
PC1	3.28 ± 0.02cd	0.63 ± 0.00cd	13.60 ± 0.31	20.92	1.48 ± 0.02d	0.60 ± 0.01d	13.40 ± 0.19	20.30
PC2	3.58 ± 0.03c	0.65 ± 0.00c	13.80 ± 0.27	19.17	1.51 ± 0.01d	0.64 ± 0.00cd	13.50 ± 0.31	20.45
PC3	3.20 ± 0.02cd	0.67 ± 0.01bc	13.60 ± 0.11	16.00	2.01 ± 0.03cd	0.67 ± 0.00c	13.30 ± 0.28	16.02
PC4	3.64 ± 0.03c	0.72 ± 0.00bc	13.70 ± 0.35	21.75	2.81 ± 0.03c	0.66 ± 0.00c	13.30 ± 0.41	22.17
PC5	6.86 ± 0.04b	0.85 ± 0.01b	14.40 ± 0.12	21.49	4.25 ± 0.02b	0.83 ± 0.02b	13.00 ± 0.26	19.40
C1	36.78 ± 0.25a	1.66 ± 0.02a	11.60 ± 0.26	6.99	20.24 ± 0.15a	1.62 ± 0.02a	11.30 ± 0.11	6.98

^‡^Values are means of three repetitions; Mean values followed by different letters are statistically different at *p* < 0.05

^a^Vitamin C (g/100 ml products)

^b^*TTA* total titratable acidity (g of malic acid/100 ml products)

^c^*TSS* total soluble solids

**Table 3:** The content of total sugar in pear and cranberrybush juice

Samples	Immediately after processing					After storage 5 m at 25 °C				
	Fructose	Sorbitol	Glucose	Sucrose	TS	Fructose	Sorbitol	Glucose	Sucrose	TS
P1	90.01 ± 0.18a^‡^	16.47 ± 0.03a	8.71 ± 0.02bc	0.85 ± 0.00a	116.03 ± 41.16A	88.85 ± 0.13a	16.69 ± 0.04	7.44 ± 0.02	0.92 ± 0.00	113.89 ± 40.77A
PC1	86.08 ± 0.34bc	15.74 ± 0.06b	9.00 ± 0.04bc	0.60 ± 0.00bc	111.42 ± 39.30BC	82.77 ± 0.23b	15.36 ± 0.04b	8.36 ± 0.01bc	0.26 ± 0.00e	106.74 ± 37.84C
PC2	86.27 ± 0.26bc	15.67 ± 0.05b	8.61 ± 0.03c	0.79 ± 0.00ab	111.33 ± 39.43BC	83.94 ± 0.20ab	14.77 ± 0.06bc	9.37 ± 0.02b	0.54 ± 0.00c	108.61 ± 38.31B
PC3	83.70 ± 0.33 cd	14.77 ± 0.05d	9.63 ± 0.03b	0.58 ± 0.00bc	108.68 ± 38.14C	83.23 ± 0.28ab	10.00 ± 0.05c	7.77 ± 0.01c	1.17 ± 0.01a	92.17 ± 40.27CD
PC4	84.91 ± 0.51c	15.06 ± 0.09bc	9.78 ± 0.07b	0.82 ± 0.00ab	110.57 ± 38.63BC	72.21 ± 0.36 cd	13.08 ± 0.04c	12.78 ± 0.04ab	0.46 ± 0.01d	98.52 ± 32.26C
PC5	87.23 ± 0.70b	16.17 ± 0.13a	8.44 ± 0.07 cd	0.64 ± 0.01b	112.48 ± 39.91B	73.72 ± 0.49c	13.29 ± 0.07c	13.11 ± 0.08a	0.52 ± 0.01c	100.64 ± 32.92C
C1	25.35 ± 0.15d	15.67 ± 0.08b	10.33 ± 0.06a	0.81 ± 0.01ab	52.16 ± 10.25D	25.47 ± 0.10d	20.47 ± 0.05a	0.81 ± 0.02d	0.85 ± 0.01b	47.59 ± 12.95D

Sugar content (g/100 ml products)

*TS* total sugar

^‡^Values are means of three repetitions; mean values followed by different letters are statistically different at *p* < 0.05

Parameters such as total soluble solids extract (TSS), total titratable acidity (TTA) and their ratio in fruit juices are commonly used in industry as quality control indices (Wojdyło et al. 2014; Visser et al. 1968).

The titratable acidity (TA) in cranberrybush was 1.66 g/100 ml and was 3.3 times higher than P1. The lower content of TA in pear juice is typical, because pear juices contain more sugar than cranberrybush, as was confirmed by other authors for quince juices (Wojdyło et al. 2014). Therefore, the total sugar (TS) of P1 was 116.03 mg/100 ml and was 2.2 times higher than C1 (Table 3). The content of TS in juices ranged from 112.48 in PC5 to 108.68 mg/100 ml in PC3. After storage the TA and TS were on average 1.3 times lower than products immediately after processing. The studies showed that the addition of cranberrybush juice did not significantly affect the decrease of the TS content in juices. Generally, a good choice to correct too sweet, too bitter or too sour taste can be mixing of different juices, which would finally lead to obtaining juices that are attractive to the consumer.

In general, the flavour intensity of pear and cranberrybush juice depends inter alia on the ratio TSS/TTA, which influences the preferences of consumers. In this research, the highest TSS/TA ratio was observed for P1 (21.21), while the lowest value was observed for C1 (6.99). In juices before and after storage, higher ratios were observed for PC1 and PC2 (average 21 and 20, respectively), while a lower value was observed for PC3 (16). Jaros et al. (2009) and Wojdyło et al. (2014) also found that the ratio TSS/TTS affects the choice and preferences of consumers in the case of cloudy apple juice and quince juice, and optimal ratios were 15:18 and 12.7:12.1, respectively. According to Jaros et al. (2009), consumers prefer sweeter juices with a higher TSS/TTA ratio. Although acidic juices are not preferred by consumers, in industry commercial juices have different requirements for acidity of juices, because this parameter affects the duration of the quality of juice. Therefore, a good solution is to mix sweet and acid juice in appropriate proportions.

### Colour parameters of juices before and after storage

Colour is one of the most decisive quality attributes of juices (Gerard and Roberts 2004). It is a parameter determining the first contact of the consumer with the product, thus shaping the desire of consumers to buy (Wojdyło et al. 2014; Mena et al. 2011). The colour parameter values, such as L*, a*, b*, ΔE*, h0*, and ΔC*, in products, before and after 5 months of storage at 25 °C, are shown in Table 4. Generally, the research showed that the colour of the products (P1, CP1-CP5, C1) differed significantly.

**Table 4:** Colour parameters of analysed juices before and after storage time (5 months at 25 °C)

Storage time and conditions	Samples	L*	a*	b*	ΔE	h°	ΔC
Immediately after processing	P1	53.11 ± 0.21a	1.22 ± 0.01f	9.77 ± 0.08a	–	–	–
	PC1	52.25 ± 0.29b	1.24 ± 0.01	9.69 ± 0.05ab	0.86	86.42	0.08
	PC2	51.50 ± 0.19bc	3.46 ± 0.04e	9.19 ± 0.07ab	2.82	4	2.31
	PC3	49.78 ± 0.19c	5.89 ± 0.07d	8.78 ± 0.07b	5.82	2.29	4.77
	PC4	49.44 ± 0.10c	8.36 ± 0.10c	8.33 ± 0.06c	8.16	2.29	7.28
	PC5	42.78 ± 0.17d	18.96 ± 0.22a	5.96 ± 0.04a	20.88	2.86	18.14
	C1	31.46 ± 0.12e	13.66 ± 0.16b	2.86 ± 0.02d	25.91	17.22	14.23
After storage 5 m at 25 °C	P1	51.63 ± 0.31ab	3.43 ± 0.04f	15.39 ± 0.10ef	6.22	81.21	6.04
	PC1	51.53 ± 0.36ab	3.99 ± 0.05 fg	15.98 ± 0.09e	6.98	78.76	6.80
	PC2	51.65 ± 0.20ab	4.40 ± 0.05e	16.68 ± 0.12d	7.75	78.04	7.61
	PC3	51.41 ± 0.42ab	4.99 ± 0.06d	17.37 ± 0.04c	8.65	76.16	8.48
	PC4	51.99 ± 0.25a	5.29 ± 0.07c	18.13 ± 0.15b	9.37	76.67	9.30
	PC5	50.66 ± 0.10c	7.34 ± 0.10a	20.71 ± 0.11a	12.77	72.65	12.54
	C1	37.26 ± 0.11d	6.01 ± 0.07b	6.09 ± 0.01 g	16.96	30.54	6.04

The value of L*, immediately after processing, ranged from 53.11 for P1 to 31.46 for C1. In general, with increasing addition of cranberrybush juice there was a decrease in brightness of pear juice. A similar situation was observed in products after 5 months of storage at 25 °C. After storage the value of L* was from 51.99 in P1 to 37.26 in C1. The lightness of the products P1 and CP1 decreased slightly, whereas in other products, L* increased.

The b* parameter values ranged from 9.77 in P1 to 2.86 in C1. Cranberrybush juice caused a change of colour to darker yellow. Moreover, storage affects the change of colour, favouring more intense yellow. The parameter after storage ranged from 20.71 in PC5 to 6.09 in C1. The value of the parameter a* in the prepared and analysed products immediately after processing was from 18.96 in juice PC5 to 1.22 in juice P1. Of course, the greater the addition of cranberrybush juice, the more parameter a* varies and the colour becomes more red. The parameter b* after storage ranged from 7.34 in juice PC5 to 3.34 in juice P1. After storage of products and the addition of cranberrybush juice to juice of pears, for example PC3, parameter b* increased from 8.78 to 17.37 and parameter a* fell from 5.89 to 4.99. Analysed products after storage are more yellow and less red.

The most important for the processing industry is parameter ΔE*. This parameter expresses the ability of the human eye to distinguish the colours of two products. It is assumed that the consumer notices a difference of colour as follows: 0 < ΔE < 1—no colour difference noted; 1 < ΔE < 2—only the experienced observer notices a difference, 2 < ΔE < 3.5—the difference is noted by the consumer; 3.5 < ΔE < 5—the consumer observes a clear colour difference between the products; 5 < ΔE—the consumer has the impression of two different colours (Pérez-Magariño and González-Sanjosé 2003). Compared with juice P1 before storage, ΔE values ranged from 0.86 in juice PC1 to 20.88 in juice PC5. After storage at 25 °C for a period of 5 months, the value of the parameter ΔE in juices ranged from 6.98 in juice P1 to 12.77 in juice PC5. After storage the value of ΔE in pear juices with the addition of cranberrybush juice, depending on the amount, increased 8.1 (PC1), 2.7 (PC2), 1.5 (PC3), and 1.1 times (PC4), and in the case of PC5 declined 0.6 times.

### Total phenolic compounds

Table 5 shows the total phenolic compounds (TPC) in P1 and C1 juices and a mixture of them. TPC in cranberrybush juice was 3823 mg/100 ml and was 4.8 times higher than pear juice. The addition of C1 significantly affects the TPC of final products. Therefore, juices with addition of C1 were characterized by TPC around 2.7, 4.0, 7.2, 2.2, and 2.6 times higher than P1. The highest concentration of TPC was observed in juice PC3, around 80% higher than juice without addition. After 5 months of storage at 25 °C, a significant (*p* < 0.05) change was observed in TPC. After storage, the TPC decreased in all products, by on average 1.2 times.

**Table 5:** The composition of phenolic compounds (TP) (mg/100 ml) and antioxidant activity (µmol Trolox/100 ml)

Samples	Immediately after processing			After storage 5 m at 25 °C		
	TP	ABTS	FRAP	TP	ABTS	FRAP
P1	773.51 ± 0.92e^‡^	458.00 ± 0.23e	382.86 ± 0.19f	646.26 ± 0.64f	361.00 ± 0.18 g	288.73 ± 0.14f
PC1	912.05 ± 1.28de	629.33 ± 0.31d	503.69 ± 0.31e	765.96 ± 0.76e	460.33 ± 0.32de	476.05 ± 0.23de
PC2	1278.42 ± 1.68d	642.33 ± 0.40de	629.48 ± 0.45d	944.49 ± 0.75d	555.17 ± 0.27e	477.33 ± 0.27d
PC3	1093.09 ± 2.89de	876.33 ± 1.00 cd	647.33 ± 0.83e	765.96 ± 0.76e	629.33 ± 0.31d	547.33 ± 0.27d
PC4	1681.06 ± 1.16c	795.33 ± 0.31c	891.85 ± 0.25c	1176.44 ± 1.17c	767.00 ± 0.15c	697.94 ± 0.34c
PC5	2891.94 ± 1.09b	1978.10 ± 0.49b	1660.13 ± 0.29b	2024.06 ± 2.02b	1152.00 ± 0.54b	1570.71 ± 0.78b
C1	3823.22 ± 3.82a	7016.67 ± 3.51a	6435.96 ± 3.22a	3176.35 ± 2.19a	6890.00 ± 1.25a	5628.67 ± 2.31a

^‡^Values are means of three repetitions; mean values followed by different letters are statistically different at *p* < 0.05

According to Sagdic et al. (2006), the content of phenolic compounds in cranberrybush was 13 199 mg/100 g and was 3.4 times higher than cranberrybush juice. The cranberrybush juice from Turkey was 1.2 times lower than our results (Sedat Velioglu et al. 2006). The total amount of polyphenol of pear juice samples varied between 196 (cv. Santa Maria) and 457 (cv. Williams) mg/100 ml and was 3.9 and 1.3 times lower than pear juice from Poland (cv. Konferencja).

### Antioxidant activity

The antioxidant activity (AA) of tested juices was measured by ABTS (free radical-scavenging activity), and FRAP (ferric reducing/antioxidant power) methods (Table 5). Results of AA in the ABTS and FRAP test showed approximately the same trends obtained by products with pears and cranberrybush, before and after storage.

Among the analysed juices before storage the highest AA in the ABTS test was determined in C1 and PC5: 7016.67 µmol Trolox/100 ml, 1978.1 µmol Trolox/100 ml, respectively. The lowest content of free radical-scavenging activity was observed in pear juice (458 µmol Trolox/100 ml). A small addition of juice cranberrybush (PC1) caused an increase in AA by 37% (PC 1) < 40% (PC 2) < 73% (PC 3). The greatest stability of AA in the ABTS test characterized the following products: C1 (98%), PC 2 (86%), C1 (79%), PC 1 (73%) and PC 3 (72%), in the case of storage for 5 months at 25 °C.

The ability to reduce iron ions determined by the FRAP assay before storage ranged from 382.86 in P1 to 6435.96 µmol Trolox/100 ml in C1. AA was higher by ca. 1.3 (PC1), 1.6 (PC 2), 1.7 (PC 3), 2.3 (PC4) and 4.3 (PC5) compared to pear juice. After storage, AA measured by FRAP methods ranged from 288.73 in P1 to 5628.67 µmol Trolox/100 ml. All products exhibited high stability of AA in the FRAP test, ranging from 75% in P1 to 95% in PC1 and PC5.

Analysis of variance showed no correlation between the potential AA and the results of organoleptic assessment. The products of the highest AA showed that they were slightly acceptable to consumers (PC5 and C1). However, the most attractive products (P1) showed lower content of AA. Moreover, products such as PC1, PC2, PC3 and PC4, which feature an average content of bioactive potential and average sensory attributes, can be good for the consumer in terms of nutritional value and quality.

### Theoretical approach for the tested products

Table 6 shows interactions between bioactive compounds in pear juice and cranberrybush juice. These interactions determine the interaction factor (IF), which estimates the power of interaction. It is a simple way of defining the type of interactions between chemical compounds or extracts in production. Their main advantage is testing between any number of compounds in the products. The proposed method requires a linear interaction between concentration and activity of a sample (Gawlik-Dziki 2012).

**Table 6:** Comparison of interaction factors (IF), of mixtures of pear and cranberrybush juice in different dose, n = 3

Samples	Immediately after processing			After storage 5 m at 25 °C		
	A_M_^†^	A_T_^¥^	IF	A_M_^†^	A_T_^¥^	IF
P1	15.77 ± 0.80a^‡^	15.77	1.00	19.30 ± 0.73	19.30	1.00
PC1	9.04 ± 0.08d	15.75	0.57	11.11 ± 0.08	19.17	0.58
PC2	4.01 ± 0.05e	15.70	0.26	4.44 ± 0.03	17.44	0.25
PC3	9.80 ± 0.63b	15.72	0.62	10.21 ± 0.21	17.01	0.60
PC4	12.73 ± 0.11c	15.60	0.82	13.53 ± 0.43	19.24	0.70
PC5	7.33 ± 0.09c	7.72	0.95	7.89 ± 0.13	17.07	0.46
C1	15.09 ± 0.00	15.09	1.00	16.82 ± 0.48	16.82	1.00

^‡^Values are means of three repetitions; mean values followed by different letters are statistically different at *p* < 0.05

^†^Measured activity (expressed as FM_50_)

^¥^Theoretical calculated activity (expressed as FM_50_)

It should be observed that PC2 juice was most active before storage. Taking into account the strength of the synergism, the mixtures of juices were arranged in the order: PC2 > PC1 ≈ PC3 > PC4 > PC5 (IF = 0.26, 0.57, 0.62, 0.82 and 0.95, respectively). In all juices the IF value was lower than 1 and a synergistic interaction was found (Table 6). After storage the synergistic interactions were similar as before storage. The storage time did not significantly affect the strength of interaction.

According to Durak et al. (2015), the IF value of a 1:1 mixture of coffee and ginger was 0.64 and showed a synergistic interaction. Furthermore, Gawlik-Dziki (2012) analysed interaction factors of mixtures of vegetables and observed that the strongest synergistic interaction was for a tomato and garlic mixture (IF = 0.11), followed by a tomato and lettuce mixture (IF = 0.68). The lowest synergistic interaction was in a tomato and onion mixture (IF = 0.80). The juices PC1 and PC3 had significantly similar synergistic interactions as tomato and lettuce, and the value of interaction in juice PC2 was similar to that in tomato and garlic.

## Conclusion

Mixtures of different doses of juices with pear and cranberrybush were characterized by high usability. Enrichment of pear juice with *Viburnum* juice had a positive impact on the improvement of the content of bioactive compounds and antioxidant properties. PC1 and PC2 were the most attractive to consumers and, what is more important, they have a good TSS/TTA ratio, which influences consumer preferences and acceptability. This ratio is commonly used in the fruit industry for quality control. Taking into account the analysis of the interaction between juices, the most active was the PC2 juice. Antioxidant activity was present in juices in various combinations, and antioxidant activity interaction seems important for their effect. In addition, interactions between antioxidants explain the efficacy of apparently low doses of active constituents. This information may be used by the juice industry as a starting point for producers of natural attractive juice mixtures.
